# Current Status of Newborn Screening in Southeastern and Central Europe

**DOI:** 10.3390/ijns12010014

**Published:** 2026-03-02

**Authors:** Nika Požun, Daša Perko, Violeta Anastasovska, Ivo Barić, Mihail Baša, Tadej Battelino, Iva Bilandžija, Ian Brincat, Miloš Brkušanin, Maja Djordević, Ivanka Dimova, Ana Drole Torkar, Ksenija Fumić, Sergiu Gladun, Panagiotis Girginoudis, Ildikó Szatmári, Ivana Kavečan, Jasmina Katanić, Vjosa Kotori, Nina Marić, Jelena Martić, Olja Manđarelo, Tatjana Milenković, Matej Mlinarič, Florentina Moldovanu, Michaela Nanu, Péter Monostori, Iskra Modeva, Branka Opančina, Dimitris Platis, Maja Raičević, Žiga Iztok Remec, Barbka Repič Lampret, Alexey Savov, Anastasia Skouma, Aleksandar Sovtić, Iva Stoeva, Alma Toromanović, Domen Trampuž, Natalia Usurelu, Jelena Višekruna, Marios Vogazianos, Maximillian Zeyda, Mojca Žerjav Tanšek, Urh Grošelj

**Affiliations:** 1 Clinical Institute for Special Laboratory Diagnostics, University Children’s Hospital, Ljubljana University Medical Center, Vrazov trg 1, 1000 Ljubljana, Slovenia; nika.pozun@kclj.si (N.P.); ziga.remec@kclj.si (Ž.I.R.); barbka.repic@kclj.si (B.R.L.);; 2 Medical Faculty, University Pediatric Clinic, P1000 Skopje, North Macedonia; violeta_anastasovska@medf.ukim.edu.mk; 3 Department of Pediatrics, University Hospital Center Zagreb, 10000 Zagreb, Croatia; ibaric@kbc-zagreb.hr; 4 Department of Pediatric Respiratory Medicine, Mother and Child Healthcare Institute of Serbia “Dr. Vukan Cupic”, 11070 Belgrade, Serbia; mihail.basa@imd.org.rs (M.B.); aleksandar.sovtic@med.bg.ac.rs (A.S.); 5 Department of Endocrinology, Diabetes and Metabolic Diseases, University Children’s Hospital, Ljubljana University Medical Center, 1000 Ljubljana, Slovenia; tadej.battelino@mf.uni-lj.si (T.B.); ana.droletorkar@kclj.si (A.D.T.); matej.mlinaric@kclj.si (M.M.); mojca.tansek@kclj.si (M.Ž.T.); urh.groselj@kclj.si (U.G.); 6 Faculty of Medicine, University of Ljubljana, 1000 Ljubljana, Slovenia; 7 Department of Laboratory Diagnostics, University Hospital Center Zagreb, 10000 Zagreb, Croatia; iva.bilandzija@kbc-zagreb.hr (I.B.); kfumic@kbc-zagreb.hr (K.F.); 8 Clinical Chemistry Laboratory, Department of Pathology, Mater Dei Hospital, MSD 2090 Msida, Malta; ian.brincat@gov.mt; 9 Centre for Human Molecular Genetics, Faculty of Biology, University of Belgrade, 11102 Belgrade, Serbia; milosb@bio.bg.ac.rs; 10 Department of Metabolism and Clinical Genetics, Mother and Child Healthcare Institute of Serbia “Dr. Vukan Cupic”, 11070 Belgrade, Serbia; maja.djordjevic@imd.org.rs; 11 Department of Medical Genetics, Medical University, 1000 Sofia, Bulgaria; idimova@medfa.mu-sofia.bg; 12 Institute of Mother and Child, MD-2062 Chisinau, Moldova; sergiu.gladun@usmf.md (S.G.); natalia.usurelu@mama-copilul.md (N.U.); 13 Department of Newborn Screening, Institute of Child Health, 11526 Athens, Greece; pgirginoudis@ich.gr (P.G.); dimitris_platis@hotmail.com (D.P.); anastasia.skouma@ich.gr (A.S.); 14 Newborn Screening and Metabolic Laboratory, Pediatric Centre, Semmelweis University, H-1085 Budapest, Hungary; szatmari.ildiko@semmelweis.hu; 15 Faculty of Medicine, University of Novi Sad, 21000 Novi Sad, Serbia; ivana.kavecan@mf.uns.ac.rs (I.K.); jasmina.katanic@mf.uns.ac.rs (J.K.); 16 Department of Genetics, Institute for Children and Youth Health Care of Vojvodina, 21000 Novi Sad, Serbia; 17 Laboratory Diagnostics Department, Institute for Children and Youth Health Care of Vojvodina, 21000 Novi Sad, Serbia; 18 Pediatrics and Clinical Laboratory Diagnostics, UBT College, Li-ori, 10000 Prishtina, Kosovo; vjosak@gmail.com; 19 Faculty of Medicine, University of Banja Luka, 78000 Banja Luka, Bosnia and Herzegovina; nina.maric@med.unibl.org; 20 University Clinical Centre of the Republic of Srpska, 78000 Banja Luka, Bosnia and Herzegovina; 21 Paediatric Department/Neonatology, Mater Dei Hospital, MSD 2090 Msida, Malta; jelena.martic@gov.mt; 22 Institute for Children’s Diseases, Clinical Centre of Montenegro, 810000 Podgorica, Montenegro; olja.mandjarelo@kccg.me (O.M.); majaraicevic89@gmail.com (M.R.); 23 Department of Endocrinology, Mother and Child Healthcare Institute of Serbia “Dr. Vukan Cupic”, 11070 Belgrade, Serbia; tanjamil5e@gmail.com; 24 National Institute for Mother and Child Health “Alessandrescu—Rusescu”, 011062 Bucharest, Romania; florentina.moldovanu@gmail.com (F.M.); nanu.micka@gmail.com (M.N.); 25 Metabolic and Newborn Screening Laboratory, Department of Pediatrics, University of Szeged, H-6720 Szeged, Hungary; monostori.peter.bela@med.u-szeged.hu; 26 Screening &Functional Endocrine Diagnostics, SBALDB “Prof. Ivan Mitev”, 1606 Sofia, Bulgaria; imodeva@abv.bg (I.M.); istoeva_p20@abv.bg (I.S.); 27 Mother and Child Healthcare Institute of Serbia “Dr. Vukan Cupic”, 11070 Belgrade, Serbia; brankaopancina@gmail.com; 28 National Genetic Laboratory, University Hospital of Obstetrics, Medical University Sofia, 1431 Sofia, Bulgaria; alexey.savov@abv.bg; 29 Faculty of Medicine, University of Belgrade, 11000 Belgrade, Serbia; 30 Department of Pediatrics, Pharmacology, Infectious Diseases, Epidemiology and Tropical Diseases, Faculty of Medicine, Burgas State University “Prof. Assen Zlatarov”, 8010 Burgas, Bulgaria; 31 Department of Pediatrics, University Clinical Center Tuzla, 75000 Tuzla, Bosnia and Herzegovina; almatoromanovic88@gmail.com; 32 Department of Pulmonology, Mother and Child Healthcare Institute of Serbia “Dr. Vukan Cupic”, 11070 Belgrade, Serbia; jelena.visekruna@imd.org.rs; 33 Center for Preventive Paediatrics “Americos Argyriou”, 3022 Limassol, Cyprus; vogazianos@cpp.org.cy; 34 Department of Pediatrics and Adolescent Medicine, Medical University of Vienna, 1090 Vienna, Austria; maximilian.zeyda@meduniwien.ac.at

**Keywords:** newborn screening, NBS, Southeastern Europe, Central Europe, neonatal screening, expanded NBS program

## Abstract

Newborn screening (NBS) is a well-established public health program that enables early detection and treatment of rare disorders in newborns, preventing severe complications or death. Despite its recognized importance, the scope and implementation of NBS programs vary across Southeastern (SE) and Central Europe. This study aimed to evaluate the current status of NBS in 16 countries of SE and Central Europe and assess progress since the previous survey in 2021. A structured questionnaire was distributed to national experts between April and December 2025, collecting data on program organization, coverage, diseases included, laboratory methods, confirmatory testing, consent practices, and future expansion plans. All countries reported universal screening for congenital hypothyroidism, except Kosovo, where a national NBS is in the process of being established. Expanded NBS using tandem mass spectrometry was available in Austria, Bulgaria, Croatia, Cyprus, Greece, Hungary, North Macedonia, Romania, and Slovenia. Spinal muscular atrophy screening became universal in Austria, Croatia, Hungary, Serbia, and Slovenia. Most countries reported plans for further expansion, with congenital adrenal hyperplasia, severe combined immunodeficiency, spinal muscular atrophy, and cystic fibrosis being the most frequently targeted conditions. Although notable infrastructural progress has been achieved, financial constraints, lack of staff, and organizational barriers remain key challenges. The study’s assessment of program effectiveness was further limited by the absence of region-wide systems for capturing end-to-end performance indicators, such as the age of the infant at treatment initiation or missed cases. Regional collaboration and adoption of best practices are therefore vital to ensure equitable access and continuous advancement of NBS programs.

## 1. Introduction

The goal of newborn screening (NBS) programs is the early detection of rare inborn disorders, ideally before symptom onset, enabling early treatment to prevent severe health complications or even death [1,2]. Screening of newborn babies was pioneered in the 1960s with screening for Phenylketonuria (PKU) by Dr. Robert Guthrie [3]. Over time, additional disorders were gradually incorporated into NBS panels, including congenital hypothyroidism (CH) in 1970, followed in the next two decades by congenital adrenal hyperplasia (CAH), hemoglobinopathies (HBPs), biotinidase deficiency (BTD), cystic fibrosis (CF), and tyrosinemia type I (TYR1) [4]. The implementation of NBS has traditionally been guided by the principles described by Wilson and Jungner, which outline 10 criteria that should ideally be met for a condition to be included in a screening program. In recent years, these criteria have been revisited and adapted to reflect advances in medicine, ethical considerations, stakeholder involvement, and the broader societal context [5].

With the implementation of tandem mass spectrometry (TMS), significant progress in the expansion of the NBS program was enabled worldwide [6]. Despite the success, many low- and middle-income countries were unable to keep pace with these advancements [7]. The development of next-generation sequencing (NGS) methods offers new opportunities to further improve and expand the NBS program, enabling earlier and more accurate diagnosis of conditions and personalized treatments, despite several remaining obstacles [8]. Furthermore, newborn genomic screening represents a promising future approach for the early detection of hundreds of monogenic disorders [9,10]. Many countries have established well-organized screening programs, which indicate that NBS represents a historically successful public health program. Although the value of NBS has been widely recognized, its introduction varies between countries due to variations in healthcare infrastructure, available resources, policy frameworks, professional engagement, and public involvement [11]. The goal of every country is to expand newborn screening to improve early detection and outcomes. This process is often limited by laboratory capacity, insufficient pilot study data, financial and technical constraints, and ethical concerns [12].

Koracin et al. (2021) conducted a survey to evaluate the status of NBS in Southeastern Europe (SE Europe) between the years 2013 and 2019 [13] following a previous survey carried out in 2014 [14]. The results indicated that, by 2019, no PKU screening had been conducted in Kosovo, North Macedonia, Malta, or Montenegro. Kosovo was planning to establish a national NBS program. Malta was the only country screening for HBP. TMS was used as a screening method in Croatia, Hungary, North Macedonia, Romania, and Slovenia. Expanded NBS was implemented in Croatia, North Macedonia, and Slovenia, and seven countries planned further expansion in the future. Expanded NBS was also implemented in Bulgaria but only as selective screening [13].

To evaluate the status of NBS in SE and Central Europe in 2024, we conducted a follow-up survey, this time also including Austria due to its geographical position (Document S1: Survey questionnaire). We mainly focused on the current status of NBS and the change, progress, and the expansion of NBS programs. In addition, we aimed to identify the laboratory methods used for first-tier and second-tier testing, determine the role of genetic testing as a confirmatory method, and examine practices related to consent and public information about NBS, as well as the procedures for sample collection, delivery, and storage.

## 2. Materials and Methods

The survey followed the same methodology as in our previous survey [13] with an adapted questionnaire to better address the fields mentioned in the previous section. In addition to the experts who responded to the first survey, we sent the questionnaire to the other NBS experts identified through the website of the International Society for Neonatal Screening (ISNS) [15]. A total of 18 questionnaires were sent to 16 countries across SE and Central Europe: Albania, Austria, Bosnia and Herzegovina (BIH) (including the entity of the Federation of BIH without Sarajevo and the entity of the Republic of Srpska), Bulgaria, Croatia, Cyprus, Greece, Hungary, Kosovo, North Macedonia, Malta, Moldova, Montenegro, Romania, Serbia (including the Autonomous Province (AP) of Vojvodina), and Slovenia. The GDP per capita for each country was obtained from the World Bank data website [16] or provided directly by the respondents. The distribution and collection of the questionnaire took place from April 2025 to December 2025, along with final clarification and data authorization by email. While the data collection and the questionnaire focused on the status of screening programs up to the end of December 2024, some important program updates that occurred in 2025 were also included. All national representatives who participated in the survey were invited to be co-authors of the study and provided authorization for the data submitted on behalf of their respective countries.

## 3. Results

### 3.1. Survey Respondents

**Figure 1:**
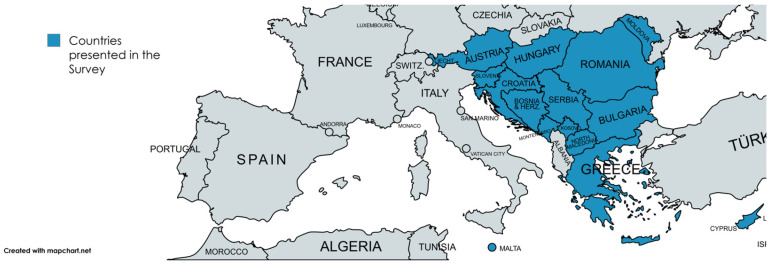
The map of SE and Central Europe with countries represented in the survey (in blue).

We received 17 responses, with Albania being the only country that did not respond. One response was obtained from each country, except for Serbia and Bosnia and Herzegovina (BIH), where more than one questionnaire was distributed due to organizational factors. In BIH, separate questionnaires were sent to the Federation of Bosnia and Herzegovina and to the Republic of Srpska (Figure 1).

### 3.2. Demographic and NBS Characteristics

Demographics and economic characteristics in SE and Central Europe are summarized in Table 1. In 2024, the total population in SE and Central Europe was approximately 76.7 million, with Romania having the largest population (19.1 million) and Malta having the lowest (563,433). The GDP per capita ranged from 6742 EUR in Kosovo to 52,552 EUR in Austria. The number of newborns ranged from 140,566 in Romania to 6964 in Montenegro.

NBS characteristics in SE and Central Europe are presented in Table 2. The number of screening centers in the country ranged from zero (Kosovo) to five (Romania). The cost of screening per newborn ranged from 0.65 EUR in Moldova to 45 EUR in Slovenia. Austria, Hungary, and Kosovo did not specify screening costs. Most of the countries reported country-wide organization of NBS, while Kosovo reported privately organized NBS programs. NBS programs were financed by the Ministry of Health and through the national health insurance schemes in most countries. Countries with the highest NBS costs, such as Slovenia and Croatia, have these costs covered through national health insurance schemes. In addition, in Austria, NBS programs were financed by the Ministry of Science, and in Kosovo by parents. In Cyprus, funding for the NBS program was approximately 50% covered by a government grant (Ministry of Health), while the rest was funded from the private sector.

### 3.3. Diseases and Laboratory Methods

All the countries implemented universal screening for CH, except for Moldova and Kosovo, where screening was not universal and was performed only in some private clinics (Table 3). Universal screening for PKU has not yet been implemented in Montenegro and Kosovo. PKU screening in North Macedonia was selective from 2013 [13] and became universal in 2024. Malta was the sole country conducting screening for hemoglobinopathies (HBPs). Additionally, cobalamin-related disorders (Cbl), including genetic Cbl defects and remethylation disorders, as well as maternally transferred vitamin B12 deficiency, were screened exclusively in Austria. Furthermore, Greece screened for cobalamin-related disorders (CblA, CblB, and CblC), which are included in the PA/MMA screening panel. Screening for spinal muscular atrophy (SMA) in 2024 was universal only in a limited number of countries, specifically Austria, Croatia, Hungary, Serbia, and Slovenia, while severe combined immunodeficiency (SCID) was screened for only in Slovenia and Austria. Additionally, in 2025, Slovenia implemented CF screening, and Greece introduced expanded NBS for several inborn errors of metabolism.

Most responders utilized the fluorescence immunoassay (FIA), and approximately half reported using tandem mass spectrometry (TMS) for NBS. High-performance liquid chromatography (HPLC) was only used in Austria, Malta, and Romania. For SMA screening, Serbia was the sole country using melting curve analysis following the polymerase chain reaction (PCR). Furthermore, colorimetric immunoassay (Cl) was only used in Austria, while North Macedonia was the sole country that used the ninhydrin method.

### 3.4. Second-Tier and Confirmatory Methods

Most responders indicated the implementation of the second-tier testing, excluding BIH—Federation of BIH (without Sarajevo), BIH—Republic of Srpska, Bulgaria, Cyprus, Kosovo, North Macedonia, Malta, and Montenegro (Table 4). For classic galactosemia (GALT), a second-tier testing FIA method was used in Austria, Sanger sequencing was applied in Greece, and the FM method was employed in Hungary. For CF, the enzyme-linked immunosorbent assay (ELISA) was used in Austria, whereas the FIA was utilized in Hungary, Romania, and Slovenia. Furthermore, in Serbia the PCR method was used for CF. The multiplex ligation-dependent probe amplification (MLPA) method served as a second-tier testing method for spinal muscular atrophy SMA in Croatia and Serbia.

Ten responders reported using genetic testing as a confirmatory method. The most frequently reported methods were next-generation sequencing (NGS), MLPA, and Sanger sequencing. Single-nucleotide (SNP) genotyping was used solely in North Macedonia. In Bulgaria, Croatia, Moldova, Serbia, and Slovenia, genetic testing was performed exclusively on-site, whereas in Hungary, North Macedonia, and Malta, testing was outsourced. Testing was conducted both on-site and through outsourcing in Greece and Romania.

### 3.5. Further Expansion

Table 5 presents data on the further expansion of NBS programs. All participating countries reported plans to expand their national NBS in the future, with the exception of Malta. Kosovo, on the other hand, is establishing a national NBS. PKU, already part of universal screening in most countries, was planned to be introduced in Montenegro in 2025. SMA, CAH, and SCID were the most frequently reported diseases to be included in the expansion plan. Kosovo was not planning expansion; instead, its main plan was to establish and begin NBS as a pilot study in 2026.

An ongoing pilot study in Moldova, introduced in 2022, uses nuclear magnetic resonance (NMR) spectroscopy to analyze newborn urine samples collected within the first week of life. Analyses are performed in collaboration with a partner institute in Iasi, Romania. This approach has allowed the additional diagnosis of three cases of methylmalonic aciduria (MMA). Notably, among the responding countries, Moldova is the only one using NMR and urine as the sample type for newborn screening.

All countries, except Montenegro, that were planning an expansion of their NBS program reported that they would conduct a pilot study prior to implementation. Countries reported that they financed pilot studies by industry sponsorship, government funding, donations, grant funding, and the Ministry of Health. The major obstacles preventing implementation of new disorders were reported as a lack of financial resources, a lack of political will, a lack of organization, a lack of staff, and low incidence. The urgency to expand the program ranged from three to five (five being the highest urgency and one the lowest), with the most frequent response being five.

### 3.6. Sample Handling

Samples for newborn screening were collected at varying ages across countries Table 6. The earliest sample collection was reported in North Macedonia, at 36–48 h after birth, while the latest occurred in Malta, at 72–120 h after birth. In most countries, samples were collected within the first 48 h of life, consistent with recommended guidelines. The average time for sample transportation from the nursery to the NBS center varied between one and six days or more. The longest transportation times were reported in BIH—Federation of Bosnia and Herzegovina (without Sarajevo) and in Moldova, where delivery required over six days. The shortest delivery times were reported in Kosovo and Malta, where samples arrived within 1 day. Samples were most commonly transported via postal or courier services. In Kosovo, samples were carried by nurses; in Malta, by hand via a liaison midwife; and in Montenegro, by hospital drivers.

All countries reported retaining samples after analysis for long-term storage following analysis. The duration of storage varied considerably, ranging from 6 months in Moldova to 24 years in Slovenia. Additionally, samples are stored lifelong in AP Vojvodina. Furthermore, in Bulgaria, samples with normal screening results were stored for five years, whereas those with pathological results were stored for at least 10 years. In most countries, dried blood spot (DBS) samples were stored in a secure institutional archive, whereas in Malta and Montenegro, samples were stored within the laboratory. Most countries reported storage at room temperature (approximately 15 –25 °C). In BIH—Federation of Bosnia and Herzegovina (without Sarajevo)—and North Macedonia, storage temperature was not controlled, whereas in Kosovo, samples were stored in a freezer at −20 °C or −80 °C. Furthermore, in Bulgaria, samples retained for 5 years were stored at room temperature, whereas those retained for 10 years were stored in a freezer (−20 °C or −80 °C).

Regarding humidity, most responders reported that it was not controlled, while in some countries it was maintained below 50%. Concerning light exposure, samples were often stored in the dark with no light exposure, while others reported limited light exposure (e.g., samples stored in opaque containers). In some cases, light conditions were not controlled.

### 3.7. Consent and Informing the Public About NBS

Most countries reported using an opt-out consent model for NBS Table 7. In contrast, only three countries—Kosovo and Romania, as well as Hungary for SMA specifically—reported the use of opt-in consent. In most countries, parents were informed about NBS after birth, primarily by a neonatologist. However, procedures varied among countries. In Austria, parents were informed before or after birth by the gynecologist, nurses, and midwives, and an information brochure was provided. In Croatia, nurses or neonatologists informed parents in maternity hospitals. In Cyprus, parents were informed after birth by the newborn’s pediatrician or neonatologist, typically immediately before the blood collection. In Hungary, information was provided during midwifery controls.

Regarding public awareness, most countries reported that the general public is informed about NBS, whereas three countries indicated that no public information activities are conducted. Additional information on how countries informed the public about NBS is presented in Table 7.

## 4. Discussion

The study evaluated the current state of NBS in SE and Central Europe, focusing on the characteristics of national programs in each country and the progress achieved since the previous survey. As an update of the 2021 study, it centered on organizational aspects of NBS—program coverage, disease panels, and laboratory methodologies—while outcome-related elements such as clinical follow-up, long-term outcomes, missed cases, and preventable complications were beyond its scope.

### 4.1. Demographics and Economics of NBS

The participating countries demonstrated substantial heterogeneity, reflecting differences not only in population size but also in economic status, as indicated by gross domestic product (GDP) per capita.

### 4.2. NBS Coverage

Rare disorders, if left untreated, can significantly reduce quality of life, cause disability, and even premature death, while also imposing a psychosocial burden on affected families. It is, therefore, essential to ensure that all newborns are tested through NBS [9]. In the majority of countries, the coverage, defined as the percentage of newborns included in neonatal screening, exceeded 95,2%, representing an improvement compared to the survey from 2013/2014 [14]. However, reported rates should be interpreted with caution as they are often based on estimates.

In Austria, the number of newborns screened in 2024 exceeded the total number of births in 2024 due to some non-residents that were screened and not counted in official birth statistics. A similar trend was observed in Slovenia as a higher number of newborns were screened than the number of newborns born due to resampled newborns and recall of positive cases. In Croatia, repeated or freshly drawn/resampled samples were included, and in Moldova, the totals did not include newborns from Transnistria. Additionally, in the case of Greece, some parents from neighboring countries give birth there to access the national NBS program.

### 4.3. Organization of NBS

The organization of NBS programs varies considerably across countries, including differences in the number of screening laboratories. According to the ISNS General Guidelines for Neonatal Bloodspot Screening (2025), the appropriate number of specimens processed by a laboratory depends on geography, the range of tests performed, and organizational and economic considerations [17].

Most countries reported having only one or two NBS centers. Romania, with the highest number of newborns (140,566), reported five screening centers. In Serbia, CH, PKU, and CF screening is performed in two regional centers: the AP Vojvodina has a single clinical center, while another center in Belgrade covers the rest of the country. In contrast, SMA screening is implemented as a centralized national programme in a third reference institution. Accordingly, three questionnaires were sent to Serbia. Austria and Greece had the largest number of samples per screening center (76,873 and 68,350), followed by Hungary (38,688), while Malta and Montenegro screened far fewer (4364 and 6964). Workload differences among countries affect the analytical quality and optimization of NBS laboratories. The high volume of samples and statistical reliability enables better performance evaluation and allows faster refinement of cut-offs and validation of methods. In contrast, low-volume centers face slower optimization, weaker statistical power, and fewer opportunities for data-driven improvements [18].

Kosovo did not establish a screening center for NBS, but it had initiated limited screening activities in some private maternity clinics. Regarding public health, this is encouraging for Kosovo, where previously there was no national NBS program. Furthermore, they also planned to establish a national program by June 2025. This represents a significant improvement compared to the previous situation, where no screening was available.

### 4.4. Cost/Economics of NBS

Cost components in economic evaluations of NBS typically include direct medical costs (screening, confirmatory testing, treatment, follow-up), direct non-medical costs (e.g., travel expenses), and indirect costs such as productivity losses. Program costs may also cover administration, quality assurance, monitoring, and tracking systems [17]. Costs ranged from the lowest 0.65 EUR in Moldova to the highest 45 EUR in Slovenia. However, it should be noted that these figures vary significantly in scope, ranging from fractional reimbursement for reagents to all-inclusive programmatic expenditures. Furthermore, since the survey did not explicitly define standardized cost-inclusion criteria, these values are not fully comparable across all participating countries and should be interpreted with caution. This challenge, in comparison, is further compounded by differences in NBS program methodology and scope. Slovenia, for example, reported the highest cost but also screens for the largest number of conditions among the countries that provided data, including SMA and SCID, which are among the most expensive disorders to incorporate into screening panels.

### 4.5. Financing of NBS

CLSI guideline NBS02 emphasizes the importance of assessing the resources available within a given geographic region for disease diagnosis, treatment, follow-up, and other necessary interventions within an NBS program. Insufficient resources can significantly limit the effectiveness and overall value of newborn screening [19]. NBS financing schemes varied considerably across countries participating in this study and differed from those in other parts of Europe. In most countries of the region, costs were covered by the Ministry of Health, the Ministry of Science, or through national health insurance schemes. In some cases—particularly during the early phases of program implementation—parents were required to cover screening costs themselves, as reported for Kosovo. In contrast, Western European countries have established more stable and standardized financing systems. In Italy, the NBS program has been financed by the government since the implementation of Law 167/2016 [9], while in Germany, screening costs are covered through national health insurance schemes [20].

### 4.6. Diseases Included in the NBS Program

When a disorder is proposed for inclusion in the NBS program, the aspects outlined in the ISNS General Guidelines for Neonatal Bloodspot Screening 2025 should be reconsidered [17]. Since the PKU was the first disease included in NBS programs, it has been successfully implemented as part of NBS in many countries for more than 50 years. However, globally, there are still countries without NBS or with an ineffective NBS program [21,22,23].

Our result showed that the only European country without screening for PKU was Montenegro, consistent with the findings reported in a 2021 survey [13], as well as Kosovo, where NBS has not yet been implemented. Several countries have successfully implemented new conditions into their NBS programs since the previous survey [13]. Screening for SMA became universal in Austria (2021), Croatia (2023), Hungary (2024), Serbia (2023), and Slovenia (2023). Screening for CF was implemented in Greece (2023), Montenegro (2024), Romania (2022), Slovenia (2025), Serbia (2009 and 2022) and Hungary (2022). Furthermore, SCID screening has been implemented only in Austria (2021) and Slovenia (2023). Additionally, Greece successfully expanded its newborn screening panel to include additional metabolic diseases in 2025. According to the answers, some diseases were still screened in a single country. For example, Greece was still the only country screening for glucose-6-phosphate dehydrogenase deficiency (G6PD). The same was applied to Malta screening for (HBP) and Austria screening for CAH and remethylation disorders (RMDs). The diseases included in the national newborn screening panel align with commonly recommended conditions described in CLSI guidelines, such as CF (CLSI NBS05), SCID (CLSI NBS06), SMA (CLSI NBS13), CAH (CLSI NBS11), and CH (CLSI NBS10) [24,25,26,27,28].

The expansion of NBS increases the number of screened conditions, but it is usually triggered by the approval of novel therapies and interventions or the discovery of new screening or diagnostic technologies. For this reason, the expansion of screening requires reconsideration of many aspects, such as economic, infrastructural, treatment-related, and ethical questions, which can delay or complicate the expansion [12,29]. According to respondents, several obstacles hindered the expansion of NBS in SE and Central Europe; however, the main reason for not achieving the goals remained a lack of financial resources, as was the case in the previous survey [13].

### 4.7. First-Tier and Second-Tier Methods

The ISNS guidelines (2025) state that for NBS, it is important to select and validate an appropriate analytical method [17]. The screening methods reported were generally consistent with those used in 2020 [13]. Austria was the only country to apply colorimetric immunoassays, and North Macedonia uniquely employed the ninhydrin method, while Serbia stood out for using PCR combined with melting curve analysis.

Screening tests are generally extremely sensitive and can detect virtually all affected individuals, but they are not necessarily very specific. This approach can increase the number of false-positive results, which can result in adding to the cost of operating NBS and cause unnecessary stress for parents. A solution to this problem could be the implementation of second-tier testing [30]. In essence, second-tier testing acts as an orthogonal screening method, aimed at refining the initial result on the same sample using a different method to improve specificity. However, it is vital to distinguish these laboratory improvements from confirmatory testing. While second-tier testing provides an additional, independent analytic layer within the laboratory workflow to enhance specificity, it remains distinct from a true clinical diagnostic test. A positive screening result, even after second-tier analysis, is considered presumptive and requires clinical validation using an independent sample—such as a sweat test for CF or specific biochemical assays—to become clinically actionable [17].

### 4.8. Genetic Testing as a Confirmatory Method

As recommended by CLSI, after any abnormal NBS result, a repeat test or confirmatory diagnostics must be performed to confirm or exclude the suspected diagnosis [19]. Our survey primarily assessed the use of genetic testing as a confirmatory method, without excluding other confirmatory approaches (e.g., enzymatic methods). Most countries reported using genetics as a confirmatory method, with some performing on-site confirmation and others outsourcing it. Resource-related challenges make it essential for a laboratory to consider whether it is better to test samples on-site or outsource them. On-site testing offers laboratories greater control over processes and enables real-time problem solving; however, it requires appropriate equipment, facilities, and skilled personnel, which can significantly increase costs. In contrast, outsourcing laboratory tests may reduce expenses, but it can compromise service reliability, extend turnaround times, and introduce potential delays that could negatively impact patient care [31].

Furthermore, various genetic methods were reported for use, such as NGS gene panels, whole exome sequencing (WES), whole genome sequencing (WGS), multiplex ligation-dependent probe amplification (MLPA), Sanger, and single-nucleotide polymorphism genotyping (SNaPshot method) [8]. In Slovenia, a pilot study was conducted in 2018 to evaluate 85 children for selected inborn errors of metabolism (IEM) using tandem mass spectrometry followed by second-tier testing, including NGS. During the study, NGS proved to be valuable in explaining the abnormal metabolite concentrations as it enabled the differentiation between affected patients and mere heterozygotes [32].

NGS technology offers several advantages, such as faster sequencing, broader sequencing range, higher sensitivity, greater accuracy, and lower cost in accordance with traditional sequencing methods such as Sanger. For that reason, NGS is often highlighted as a promising method also for the first tier in NBS. However, despite its potential in the expansion of NBS, several concerns, such as technical feasibility, economic and medical considerations, legal, ethical, and psychological implications, need to be addressed [8]. Furthermore, in cases of a negative or inconclusive genetic result with persistently abnormal biochemical findings, further diagnostic evaluation using complementary genetic or functional methods remains clinically indicated.

### 4.9. Sample Collection and Transportation

Properly collected and transported DBS is an important step in early detection and treatment in case of a diagnosis [33]. The survey results showed that samples were typically collected 48–72 h after birth. Timing of collection is important because some metabolite and hormone levels vary markedly in the neonatal period, which can impact screening performance and results. Collecting too early (before 48 h) may miss some metabolic disorders, while collecting too late (after 48 h) could delay diagnosis and treatment [34]. Screening for primary CH in early samples may lead to false-positive results due to the physiological postpartum thyrotropin surge. For this reason, it is recommended that samples be taken not before 24 h after birth [35]. The opposite applies for PKU, where samples taken too early (before 48 h) can lead to false-negative results as phenylalanine blood concentrations can be lower and yet stabilized [36].

According to CLSI guideline NBS01, NBS programs currently recommend initial specimen collection at 24 to 48 h of age [37]. This difference, observed in our results, is mainly due to longer postpartum hospital stays in SE and Central European countries (usually 3–4 days), allowing for later sampling without the risk of missing the infant before discharge [18].

The Clinical Laboratory Standards Institute (CLSI) recommends that DBS collected from a baby’s heel prick should be transported to the testing facility as soon as the DBS are dry (a minimum of 3 h) and no later than 24 h after collection, regardless of weekends [37]. Based on survey responses, transportation delays to the laboratory should be minimized. A recent case report highlighted that early lethal presentation of MTPD/LCHADD and other FAODs underscores the need for rapid processing of NBS samples. However, even timely screening cannot always prevent fatal outcomes as disease onset can occur within hours or days after birth, making early intervention challenging [38]. An international survey of late-diagnosed (PKU) cases showed that undiagnosed cases can occur everywhere, even in countries with well-established screening programs, if individuals are missed due to migration or systemic gaps [21]. This highlights that the effectiveness of NBS depends not only on technology but also on accessibility and population coverage.

### 4.10. Sample Storage

According to CLSI guideline NBS01, extended storage and use of residual DBS specimens can be valuable for quality control and NBS program development and have proven valuable for expanding NBS panels to include additional conditions [37]. In our survey, most countries reported extended storage (two years or more). Three of them (Austria, Greece and Kosovo) reported low humidity levels (less than 50%), and only in Kosovo were DBS samples stored under sub-zero conditions. Most countries in our survey reported that they stored DBS in a secure archive within the institution. Our results indicate that compliance with these recommendations varies across laboratories. Additionally, CLSI guideline NBS01 recommends that residual DBS specimens stored for extended periods should be protected to ensure specimen integrity, for example, by using low gas–permeable plastic bags with desiccant and maintaining storage at ≤−20 °C for periods longer than two years [37]. In contrast, respondents reported storing samples for extended periods at room temperature, which is not in accordance with the guidelines. This practice is likely influenced by financial constraints, such as the cost of refrigeration equipment or limited storage space.

### 4.11. Consent and Informing About NBS

Consent for NBS is complex and varies internationally. Approaches range from opt-out models, where newborns are automatically included unless parents actively decline, to opt-in systems that require explicit parental agreement to participate [39]. Most respondents reported using an opt-out approach, which generally results in higher screening coverage. This is beneficial for the NBS itself as higher participation ensures more equitable access, improves early detection of rare conditions, and enhances the overall public health impact of NBS.

Informing parents about NBS is an important part of the NBS program as it promotes trust and fosters support for NBS. Furthermore, it enables parents to make an informed decision about participation [40]. During the prenatal period, families need generalized NBS information that broadly answers questions such as: 1. What is NBS? 2. Why is NBS important? 3. How is NBS performed? 4. How do families receive NBS results? 4. What will happen if the screening is positive? 5. Who can families contact if they have questions? [19]. All countries reported informing parents about NBS; most of them reported informing after birth. A randomized clinical trial in the USA showed that prenatal education with the use of multimedia tools and electronic platforms could have positive effects on parents’ awareness and knowledge about NBS [41]. In addition, our survey indicates that several countries informed the public about NBS via multimedia tools and a website-based platform, which could also be part of prenatal education for parents. Our results are consistent with CLSI guideline NBS02, which recommends that parents, healthcare professionals, and the public be aware of the NBS program and understand the screening process [19].

### 4.12. Limitations and Future Work

While our survey highlights significant progress in NBS programs across the region, it has certain limitations. The study primarily mapped organizational structure, screening panels, laboratory approaches, and coverage but did not assess the full NBS continuum. Specifically, it did not include detailed data on clinical follow-up, parental compliance, the coordination and funding of confirmatory testing, or long-term clinical outcomes.

Furthermore, key performance indicators—such as the age of the infant at treatment initiation and the rate of missed cases—remain unassessed [17]. We recognize that such granular, population-based data are often challenging to obtain due to fragmented reporting systems. However, these metrics are essential for evaluating the public health and economic impact of expanded screening. Future region-wide efforts should focus on capturing this end-to-end performance to provide the evidence needed for sustainable funding and to ensure equitable, effective care for all newborns.

## 5. Conclusions

The results of this survey demonstrate that newborn screening (NBS) programs in SE and Central Europe have made progress in terms of infrastructure and programmatic expansion over the past four years, although substantial differences between countries remain. Coverage rates exceeded 99% in most countries, marking an improvement compared to previous surveys. Several countries introduced new conditions into their national programs, most notably SMA, CF, and expanded panels for metabolic disorders. This progress mainly reflects the expansion of disease panels and, importantly, the development of screening programs in countries where such screening was previously absent or limited to only a few conditions. However, challenges such as limited financial resources, workforce shortages, and organizational barriers continue to hinder further expansion. Most countries plan to broaden their programs, with CAH, SCID, SMA, and additional metabolic disorders being the most frequently targeted conditions. Key priorities include improving laboratory quality, reducing false-positive results through second-tier testing, and strengthening parental and public awareness. Moving forward, regional collaboration, sharing of best practices, and the development of standardized guidelines will be essential to ensure equitable access and continuous improvement of NBS programs across the region.

## Figures and Tables

**Table 1:** Demographics and economic characteristics in Southeastern and Central Europe.

Country	Total Population	GDP per Capita in 2024	Number of Newborns in 2024	Number of Newborns Screened in 2024	Coverage in 2024
Austria	9.1 million	52,552 EUR	76,873	77,820 ^1^	N/A
BIH—Federation of BIH (without Sarajevo)	1.3 million	8145 EUR	11,618	11,591	99.7%
BIH—Republic of Srpska	1.1 million	7158 EUR	9150 ^2^	9150 b ^2^	100% ^3^
Bulgaria	6.4 million	16,066.3 EUR	53,428 ^4^	50,099 ^1^	N/A
Croatia	3.9 million	22,115 EUR	32,069	32,521 ^5^	N/A
Cyprus	1 million	35,714 EUR	9900 d ^6^	9865	99.64%
Greece	9.9 million	22,881 EUR	68,350	68,350 ^7^	100% ^3^
Hungary	9.6 million	21,538 EUR	77,511	77,376	99.84%
Kosovo	1.5 million	6742 EUR	21,368	/	/
North Macedonia	1.8 million	8602 EUR	16,019	15,869	99%
Malta	0.57 million	39,133 EUR	4374	4364	99%
Moldova	2.4 million	7037 EUR	23,862 ^8^	23,108 ^7^	99%
Montenegro	0.62 million	11,950 EUR	6964	6960	99.94%
Romania	19.1 million	18, 543 EUR	140,566	133,841 ^9^	95.2%
Serbia	6.6 million	12,493 EUR	60,845	60,310	99.12%
Slovenia	2.1 million	31,493 EUR	16,875	17,332 2	N/A

^1^ Some non-residents were screened and are not included in official birth statistics. ^2^ The number is estimated. ^3^ Value is approximate due to estimated data. ^4^ Including 3714 born outside Bulgaria. ^5^ Repeated or recollected/new samples from the same newborn are included. ^6^ The government has not yet released the official number. ^7^ Since there is no registry linked to the national NBS, this number is not accurate; however, NBS is universal. Some parents from neighboring countries deliver in Greece to access the national NBS program. ^8^ Excludes Transnistria. ^9^ The number does not include newborns who underwent expanded screening for additional conditions through private services outside the national program. Abbreviations: N/A—Not applicable, /—Data not available.

**Table 2:** NBS characteristics in SE and Central Europe.

Country	Number of Screening Centers in 2024	Estimated Cost or Reimbursement per Newborn ^1^	Organization of NBS	NBS Financing
Austria	1	/	Country-wide	By the Ministry of Science
BIH—Federation of BIH (without Sarajevo)	1	18 EUR	Country-wide	Through the national health insurance schemes
BIH—Republic of Srpska	1	7.5 EUR	Country-wide	Through the national health insurance schemes
Bulgaria	2 ^2^	Direct costs ^3^: PKU = 1.4 EUR, CAH 2.14 EUR, TSH 1.4 EUR, FPC 0.5 EUR ~6 EUR	Country-wide	By the Ministry of Health (reagents), NHIF, Charities (some instruments)
Croatia	1	32.73 EUR	Country-wide	Through the national health insurance schemes
Cyprus	1	24 EUR	Country-wide	Up to approximately 50% covered by a government grant (Ministry of Health), and the rest is from the private sector.
Greece	1	8 EUR	Country-wide	By the Ministry of Health
Hungary	2	/	Country-wide	By the Ministry of Health Through the national health insurance schemes
Kosovo	0 ^4^	/	Private	By parents
North Macedonia	1	CH = 2.8 EUR, CF = 2.8 EUR PKU = 1.6 EUR, other metabolic diseases = 14.5 EUR	Country-wide ^5^	By the Ministry of Health
Malta	1	15 EUR ^6^	Country-wide	By the Ministry of Health
Moldova	1	0.65 EUR	Country-wide	By the Ministry of Health
Montenegro	1	10 EUR	Country-wide	By the Ministry of Health
Romania	5	PKU = 2.5 EUR, CH = 2.5 EUR CF = 5 EUR	Country-wide	By the Ministry of Health
Serbia	2 for CH, PKU, and CF 1 for SMA	CF, CH, and PKU = 20 EUR SMA = 7.91 EUR	Country-wide	Through the national health insurance schemes
Slovenia	1	45 EUR	Country-wide	Through the national health insurance schemes

^1^ Cost data were reported by national representatives based on local availability and may range from direct laboratory costs to full program expenditures. Since the survey did not explicitly define cost-inclusion criteria, these values are not fully comparable across all participating countries. ^2^ At the University Obstetric Hospital, all newborns are screened for phenylketonuria (PKU). At SBALDB I. Mitev, all newborns are screened for congenital hypothyroidism (CH) and congenital adrenal hyperplasia (CAH). ^3^ Cost of reagents covered by Ministry of Health. ^4^ In Kosovo, there is no NBS program. Kosovo was in the beginning stage of starting the NBS program by June 2025. Currently, NBS is organized in some maternity private clinics, and testing for CH is carried out by a considerable number of pediatricians on day five. ^5^ CH, CF, and PKU are part of NBS, while screening for metabolic diseases is selective for 6000 newborns. ^6^ This covers only consumable costs; human resources not included. Abbreviations: SMA—Spinal muscular atrophy, /—Data not available, NHIF—National Health Insurance Fund.

**Table 3:** Diseases and laboratory methods.

Country	Diseases for Which Screening Is Universal (Year of Introduction)	Laboratory Methods for NBS
Austria	ARG (2017), BTD (1984), CAH (1997), CH (1987), CF (1997), CIT1 (2002), CIT2 (2002), Cbl ^1^ (2017), CPT1 (2002), CPT2 (2002), CUD (2002), GA1 (2002), GA2 (2002), GALT (/), HCY (2002), H-PHE (1968), IVA (2002), LCHADD (2002), MCADD (2002), MET (2002), MSUD (2002), PA/MMA (2002), PKU (1966), RMDs (2017), SCID (2021), SMA (2021), TYR (2002), VLCADD (2002)	FM, FIA ^2^, HPLC, TMS, qPCR, RT-PCR, CI
BIH—Federation of BIH (without Sarajevo)	CH-(2000, 2005) ^3^, PKU (2001, 2005) ^4^	FIA
BIH—Republic of Srpska	CH (2007), PKU (2007)	FIA
Bulgaria	CAH (2010), CH (1993), PKU (1978/79), Galactosemia (1989–1993 discontinued), selective extended metabolic screening since 2009/10 by TMS	FIA, FM, TMS
Croatia	CH (1985), CUD (2017), GA1 (2017), H-PHE (1978), IVA/2-MBG (2-MBG is not a primary target of NBS) (2017), LCHADD (2017), MCADD (2017), PKU (1978), VLCADD (2017), SMA (2023)	FIA, TMS, RT-PCR
Cyprus	CH (1989), PKU (1989)	FM, FIA, TMS,
Greece	CH (1979), GALT (2006), PKU (1974), G6PD, H-PHE (1974), CF (2023), CUD (2025), GA1 (2025), GA2 (2025), PA/MMA ^5^ (2025), IVA (2025), VLCADD (2025), MCADD (2025), LCHADD (2025), MSUD (2025), TYR1 (2025), 3MCC (2025), CPT1 (2025), CPT2 (2025), SCAD (2025), 3HMG (2025), HSD (2025), BKT (2025), MAL (2025), ARG (2025), CIT1 (2025), CIT2 (2025)	FM, FIA, TMS
Hungary	BTD (1990), CH (1985), CF (2022), CIT1 (2007), CPT1 (2007), CPT2 (2007), CUD (2007), GA1 (2007), GA2 (2007), GALT (1975), HCY (2007), H-PHE (2007), IVA (2007), LCHADD (2007), MCADD (2007), MSUD (2007), PA/MMA (2007), PKU (1975), SCAD (2007), TYR1 (2007), VLCADD (2007), 3HMG (2007), BKT (2007), 3MCC (2007), MCD (2007), SMA (2024) ^6^	FM, FIA, TMS, RT-PCR
Kosovo	CH—However, screening is not universal and is only performed in some private maternity clinics. Almost all testing has been conducted abroad, with samples being sent outside Kosovo for nearly five years.	/
North Macedonia	Universal screening for diseases: CH (2007), CF (2019), PKU (2024). Selective screening for diseases (since 2013): CIT1 (2013), CIT2 (2013), CPT1 (2013), CPT2 (2013), CUD (2013), GA1 (2013), HCY (2013), H-PHE (2013), IVA (2013), MAL (2013), MCADD (2013), MET (2013), MSUD (2013), PA/MMA (2013), SCAD (2013), TYR1 (2013), BKT (2013), ARG (2013), VLCADD (2013), 3HMG (2013), BKT (2013), 3MCC (2013), MCD (2013)	FIA, TMS, Ninhydrin method
Malta	CH (1989), HBP (1989), PKU (2020)	FM, FIA, HPLC
Moldova	H-PHE (2016), PKU (1989)	FM
Montenegro	CH (2007), CF (2024)	FM
Romania	CH (2010), PKU (2010), CF (2022)	FM, FIA -DELFIA, HPLC, TMS
Serbia	CH (1983, 2003) ^7^, CF (2009, 2022) ^7^, PKU (1983, 2000) ^7^, SMA (2023),	RT-PCR, PCR, melting curve analysis, DELFIA, FM
Slovenia	CH (1981), PKU (1979), CUD (2018), GA1 (2018), GA2 (2018), PA/MMA (2018), IVA (2018), VLCADD (2018), MCADD (2018), LCHADD (2018), MSUD (2018), TYR1 (2018), 3MCC (2018), CPT1 (2018), CPT2 (2018), 3HMG (2018), HSD (2018), BKT (2018), SMA (2023), SCID (2023), CF (2025)	FIA, TMS, qPCR, RT-PCR

^1^ Cobalamin-related disorders (Cbl) including genetic Cbl defects and remethylation disorders as well as maternally transferred vitamin B12 deficiency. ^2^ Including DELFIA. ^3^ 2000 in Tuzla Canton. ^4^ 2005 in the Federation of Bosnia and Herzegovina (except Sarajevo). ^5^ Including cobalamin-related defects (CblA, CblB, CblC). ^6^ SMA screening is performed only following a parental informed consent (opt-in), and it is not mandatory. ^7^ Two regional centers initiated screening for the listed disease at different times. Abbreviations: ARG—Arginase deficiency, BKT—β-ketothiolase deficiency, BTD—Biotinidase deficiency, CAH—Congenital adrenal hyperplasia, CH—Congenital hypothyroidism, CF—Cystic fibrosis, CIT1—Citrullinemia type 1, CIT2—Citrullinemia type 2, Cbl—Cobalamin- related disorders, CPT1—Carnitine palmitoyltransferase deficiency type 1, CPT2—Carnitine palmitoyltransferase deficiency type 2, CUD—Carnitine uptake defect, G6PD—Glucose-6-phosphate dehydrogenase deficiency, GA1—Glutaric acidemia type 1, GA2—Glutaric acidemia type 2, GALT—Classic galactosemia, HBP—Haemoglobinopathy, 3HMG—3-Hydroxy-3-methylglutaric aciduria, HCY—Homocystinuria, H-PHE—Hyperphenylalaninemia, HPTI—Hypoxanthine-guanine phosphoribo-syltransferase deficiency, HSD—Holocarboxylase synthetase deficiency, IVA—Isovaleric acidemia, 2-MBG—2-Methylbutyrylglycinuria, LCHADD—Long-chain 3-hydroxyacyl-CoA dehydrogenase deficiency, 3MCC—3-Methylcrotonyl-CoA carboxylase deficiency, MCD—Multiple carboxylase deficiency, MET—Hypermethioninemia, MSUD—Maple syrup urine disease, MAL—Malonic acidemia, MCADD—Medium-chain acyl-coenzyme A dehydrogenase deficiency, PA/MMA—Propionic/methylmalonic aciduria, PKU—Phenylketonuria, RMDs—Remethylation disorders, SCAD—Short-chain acyl-CoA dehydrogenase deficiency, SCID—Severe combined immunodeficiency, SMA—Spinal muscular atrophy, TYR1—Tyrosinemia type 1, VLCADD—Very long-chain acyl-CoA dehydrogenase deficiency, CI—Colorimetric Immunoassays, DELFIA—Dissociation-enhanced lanthanide fluorescence immunoassay, FM—Fluorometric method, FIA—Fluorescence immunoassay, HPLC—High-performance liquid chromatography, TMS—Tandem mass spectrometry, qPCR—Quantitative PCR (applies to SCID), RT-PCR—Real-time PCR (applies to SMA), /—Data not available.

**Table 4:** Second-tier testing and confirmatory methods.

Country	Second-Tier Testing in Screening	Laboratory Methods for Second-Tier Testing (With Disease Indications)		Genetic Testing as a Confirmatory Method ^1^	Method of Genetic Testing (Type of Confirmation)
		Laboratory Methods	Diseases		
Austria	Yes	HPLC-TMS-coupled	HCY, MMA, PA, Cbl, RMD	No	/
		FIA	GALT		
		ELISA	CF		
BIH—Federation of BIH (without Sarajevo)	No	/	/	No	/
BIH—Republic of Srpska	No	/	/	No	/
Bulgaria	No ^2^	/	/	Yes	Sanger, NGS gene panel, WES, MLPA (on-site)
Croatia	Yes	MLPA	SMA	Yes	NGS gene panel, MLPA (on-site)
Cyprus	No	/	/	No	/
Greece	Yes	Sanger	GALT	Yes	Sanger, NGS panel, MLPA (on-site); WES (outsourced)
Hungary	Yes	TMS	TYR	Yes	Sanger, MLPA, NGS gene panel (outsourced)
		FIA	CF		
		FM	GALT		
Kosovo	No	/	/	No	/
North Macedonia	No	/	/	Yes	Sanger, NGS gene panel, WES, WGS, SNP genotyping (SnaPshot, outsourced)
Malta	No	/	/	Yes	Sanger, NGS gene panel (outsourced)
Moldova	Yes	HPLC	PKU	Yes	Sanger (on-site) ^3^
Romania	Yes	HPLC	PKU	Yes	NGS gene panel (on-site for CF, but for PKU only in some of the screening centers), NGS whole genome (outsourced)
		FIA	PKU, CF		
		TMS	PKU		
Montenegro	No	/	/	No	/
Serbia	Yes	PCR MLPA	CF, PKU, SMA	Yes	PCR, NGS gene panel, MLPA (on-site)
Slovenia	Yes	FIA	CF	Yes	NGS gene panel, WES, WGS, MLPA (on-site)

^1^ Does not exclude the use of other confirmatory methods (e.g., enzymatic methods). ^2^ The plan is to implement as second tier for CAH from the 1st FPC by steroid profiling TMS. ^3^ The PCR analysis of the most frequent 5 mutations of the PAH gene is performed first, and then if the molecular–genetic results are negative but high Phe level persists, the Sanger analysis of the PAH gene is performed. /—Data not available.

**Table 5:** Further expansion.

Country	Plans for Further Expansion	Diseases Included in the Further Expansion Plan (Year of Introduction)	Conducting a Pilot Study Before Proceeding with Expansion	Financing Pilot Studies	The Main Reasons That Prevent Further Expansion	Urgency Rate for Expanding NBS (1—Lowest Rate, 5—Highest Rate)
Austria	Yes	HBP (2026) LSD (/)	Yes	Industry sponsorship	Lack of financial resources, lack of political will	4
BIH—Federation of BIH (without Sarajevo)	Yes	CF (/) SMA (/)	Yes	Industry sponsorship	Lack of financial resources, lack of organization, lack of political will	5
BIH—Republic of Srpska	Yes	SMA (2025)	Yes	Government funding	Lack of financial resources	4
Bulgaria	Yes	SMA, SCID, CF (2026) Several other metabolic diseases by TMS	Yes	Government funding, grant funding	Lack of staff, lack of financial resources	5
Croatia	Yes	HCY (2025) Several others (/)	Yes	Government funding, industry sponsorship	Lack of financial resources, lack of staff	3
Cyprus	Yes	CAH, GALT, G6PD, MSUD, HCY, GA1, IVA, MCADD (2026)	Yes	Government funding, donations	Lack of financial resources, lack of political will, other ^1^	5
Greece	Yes	CAH, BTD (2027), SCID, SMA (2026)	Yes	Government funding, grant funding	Lack of financial resources, lack of organization, lack of political will	5
Hungary	Yes	CAH (pilot drafting), SCID (waiting for pilot approval)	Yes	Government funding	Lack of financial resources	5
Kosovo	No, but the public sector will begin the NBS as a Pilot study in 2026.	CF, GALT, PKU, SMA, and CH (2026)	Yes	Ministry of Health	Lack of financial resources Lack of political will	5
North Macedonia	Yes	SCID, SMA (2025)	Yes	Government funding, grant funding	Lack of financial resources Low incidences	3
Malta	/	/	/		Lack of staff Lack of organization	4
Moldova	Yes	Ongoing pilot-NBS by NMR spectroscopy of the urine of newborns from IMC’s maternity (2025), Expanded screening by TMS, for a minimum of 15 diseases, including PKU (2027), CH (2027), CF (2028), GALT (2028). SMA (on-going pilot-screening), SCID (pilot from 2026)	Yes	Institutional funding grant funding, research collaborations	Lack of financial resources	5
Montenegro	Yes	CAH (2025), PKU (2025)	No	/	Lack of financial resources Lack of staff	5
Romania	Yes	GALT, CAH, SMA, SCID (/)	Yes	Industry sponsorship, grant funding	Lack of financial resources Lack of staff Lack of organization	5
Serbia	Yes	CAH (2026), BTD, GALT SCID, G6PD, and XLA (Projected for 2027), MCADD, VLCADD, LCHADD, CUD, GA1, IVA, PA, MMA, MSUD	Yes	Industry sponsorship, grant funding Government funding	Lack of financial resources	5
Slovenia	Yes	CAH (pilot completed with implementation planned for early 2026), CMV (/)	Yes	Grant funding, research projects	Lack of financial resources Lack of staff	4

^1^ Due to bureaucracy and a lack of political will, the new expansion panel—proposed in 2016 by a committee of specialists and approved six years later by the Council of Ministers—has still not been implemented. Abbreviations: BTD—Biotinidase deficiency, CAH—Congenital adrenal hyperplasia, CF—Cystic fibrosis, CH—Congenital hypothyroidism, CMV—Cytomegalovirus, CUD—Carnitine Uptake Deficiency, G6PD—Glucose-6-phosphate dehydrogenase deficiency, GALT—Galactosemia, GA1—Glutaric acidemia type 1, HBP—Haemoglobinopathy, HCY—Classical homocystinuria, IVA—Isovaleric acidemia, LCHADD—Long-chain hydroxyacyl-CoA dehydrogenase deficiency, LSD—Lysosomal storage disorders, MCADD—Medium-chain acyl-CoA dehydrogenase deficiency, MMA—Methylmalonic acidemia, MSUD—Maple syrup urine disease, PA—Propionic academia, PKU—Phenylketonuria, SCID—Severe combined immunodeficiency, SMA—Spinal muscular atrophy, XLA—X-linked agammaglobulinemia, NMR—Nuclear magnetic resonance, TMS—Tandem mass spectrometry, VLCADD—Very long-chain acyl-CoA dehydrogenase deficiency, /—Data not available.

**Table 6:** Sample collection, delivery, and storage.

Country	Age at Blood Collection Date	Average Transport Time (Nursery → NBS Center)	Sample Transport Personnel	Samples Stored Long-Term	Storage Duration	Storage Location	Storage Temperature	Storage Humidity	Storage Light Level
Austria	36–72 h	2 days	Post office/mail carrier Courier service	Yes	10 years	Secure archive within the institution	RT	Less than 50%	Stored in the dark
BIH—Federation of BIH (without Sarajevo)	72–96 h	over 6 days or more	Post office/mail carrier	Yes	10 years	Secure archive within the institution	Not controlled	Not controlled	Stored in the dark
BIH—Republic of Srpska	48–96 h	4 days	Post office/mail carrier Courier service	Yes	10 years	Secure archive within the institution	RT	Not controlled	Limited light exposure
Bulgaria	48–120 h	2–5 days	Courier service	Yes	5 years and min. 10 for path. result	Secure archive within the institution	5 years—RT 10 years—FT	Not controlled	Stored in the dark
Croatia	48–72 h	5 days	Post office/mail carrier Courier service	Yes	5 years	Secure archive within the institution	RT	Not controlled	Limited light exposure
Cyprus	48–72 h	1–2 days	Courier service	Yes	2 years	Secure archive within the institution	RT	Partly controlled	Limited light exposure
Greece	48–72 h	3 days	Courier service	Yes	10 years	Secure archive within the institution	RT	Less than 50%	Stored in the dark
Hungary	48–72 h	4 days	Post office/mail carrier	Yes	3–5 years	Within institution	RT	Not controlled	Limited light exposure
Kosovo	72 h	1 day	Nurses	Yes	Two weeks	Secure archive within the institution	FT	Less than 50%	Limited light exposure
North Macedonia	36–48 h	4 days	Post office/mail carrier Courier service	Yes	3 years	Secure archive within the institution	Not controlled	Not controlled	Stored in the dark
Malta	72–120 h	1 day	By hand by the discharge liaison midwife	Yes	No retention policy; cards from last 4 years stored	Within the NBS lab	RT	Not controlled, Desiccants are added to each plastic with DBS.	Stored in the dark
Moldova	After 48 h	Over 6 days or more	Post office/mail carrier Courier service	Yes	6 months	Secure archive within the institution	RT	Not controlled	Stored in the dark
Montenegro	48–72 h	5 days	Hospital drivers during patient transport to the hospital	Yes	1 Year	Secure in the laboratory	RT	Not controlled	Limited light exposure
Romania	48 h	5 days	Courier service	Yes	5 years	Secure archive within the institution	RT	Not controlled	Not controlled
Serbia	48–72 h	2 days	Post office/mail carrier Courier service	Yes	5 years; AP Vojvodina: lifelong	Secure archive within the institution	RT	Not controlled	Stored in the dark
Slovenia	48–72 h	3 days	Post office/mail carrier Courier service	Yes	24 years	Secure archive outside the institution	RT	Not controlled	Stored in the dark

Abbreviations: AP—autonomous province.

**Table 7:** Consent and informing the public about NBS.

Country	Type of Consent	Informing Parents About NBS	Informing the Public About NBS	Type of Informing
Austria	Opt-out	Before or after birth, by the gynecologists, nurses, and midwives.	Yes	Website, brochures, and leaflets ^1,2^
BIH—Federation of BIH (without Sarajevo)	Opt-out	After birth by the neonatologist.	Yes	Website, brochures, and leaflets
BIH—Republic of Srpska	Opt-out	After birth by the neonatologist	Yes	Through media
Bulgaria	Opt-out	Before and mainly after birth by the neonatologist.	Yes	Brochures and leaflets, Official site of the Ministry of Health ^3^, Regulation No26/2007 of the MoH ^4^
Croatia	Opt-out	staff (nurses or neonatologists) in maternity hospitals	Yes	Website ^5,6,7^
Cyprus	Opt-out Inferred	After birth by the newborn’s pediatrician (or neonatologist) before the blood collection.	Yes	Website ^8^, brochures and leaflets, events organized by CPP or by volunteers, through several activities, social media, mass media, etc.
Greece	Opt-out	After birth, by the neonatologist	Yes	Website ^9^, brochures and leaflets
Hungary	Opt-out Opt-in (SMA)	During midwifery controls	Yes	Brochures and leaflets ^10^, district nurse
Kosovo	Opt-in— oral	After birth, by the neonatologist or by the pediatrician	Yes	Media campaign, TV and radio
North Macedonia	Opt-out	After birth, by the neonatologist	No	/
Malta	Opt-out	After birth, by the neonatologist	No	/
Moldova	Opt-out	After birth, by the neonatologist	Yes	Brochures and leaflets, by parents of Association of PKU children, by TV
Montenegro	Opt-out	After birth by the neonatologist	No	/
Romania	Opt-in— Written	After birth by the neonatologist	Yes	Website ^11^
Serbia	Opt-out	After birth by the neonatologist	Yes	Website ^12,13^, frequent media appearances
Slovenia	Opt-out	After birth by the neonatologist	Yes	Website ^14^

^1^ https://kinder-jugendheilkunde.meduniwien.ac.at/unsere-abteilungen/klinische-abteilung-fuer-paediatrische-pulmologie-allergologie-und-endokrinologie/neugeborenen-screening/ (accessed on 25 December 2025). ^2^ https://kinder-jugendheilkunde.meduniwien.ac.at/fileadmin/content/OE/kinder-jugendheilkunde/Diverse/neugeborenenscreening/NGS_Folder_2022-v17.pdf (accessed on 25 December 2025). ^3^ https://www.mh.government.bg/bg/novini/aktualno/4494 (accessed on 17 December 2025). ^4^ https://lex.bg/laws/ldoc/2135556407 (accessed on 17 December 2025). ^5^ Informacije javnosti o novorođenačkom probiru Klinički bolnički centar Zagreb. ^6^ https://www.kbc-zagreb.hr/EasyEdit/UserFiles/informacije-za-lijecnike/informacije-javnosti-o-novorodjenackom-probiru-2023-03-01.pdf (accessed on 17 December 2025). ^7^ https://www.hzjz.hr/wp-content/uploads/2023/03/Informacije-za-javnost-o-novorodenackom-probiru-2023.pdf (accessed on 17 December 2025). ^8^ https://www.cpp.org.cy/en/programma-proliptikou-elegxou-neognon (accessed on 17 December 2025). ^9^ www.eppen.gr. ^10^ https://semmelweis.hu/bokayklinika/betegellatas/diagnosztikai-egysegeink/anyagcsere-szuro-es-diagnosztikai-kozpont/, https://u-szeged.hu/szakk/anyagcsere (accessed on 17 December 2025). ^11^ www.insmc.ro. ^12^ https://dnkanaliza.rs/neonatalni-skrining/ (accessed on 17 December 2025). ^13^ https://www.izzzdiovns.rs/. ^14^ https://www.redkebolezni.si/.

## Data Availability

Data are available upon request.

## Supplementary Materials

The following supporting information can be downloaded at: https://www.mdpi.com/article/10.3390/ijns12010014/s1, Document S1: Survey questionnaire.
