# Supplementary material for: Current Status of Newborn Screening in Southeastern and Central Europe
Source: Int J Neonatal Screen. 2026 Mar 2;12(1):14. doi: 10.3390/ijns12010014 (PMC13026941; doi:10.3390/ijns12010014)
Supplement: Supplementary file 1 [file IJNS-12-00014-s001.zip › IJNS-4100511-supplementary.pdf]

Questionnaire: Current Status of Newborn Screening in Southeastern and Central Europe.

Dear colleagues,

We kindly invite you to participate in a survey conducted by the University Children's Hospital Ljubljana, to explore the current state of the newborn screening (NBS) programs in the region of Southeastern/Central Europe.

The study's purpose is to assess the current state of NBS programs in the region and compare it to the data obtained from the last two surveys in 2014 and 2021 (10.1016/j.ymgme.2014.07.020; 10.3389/fped.2021.648939).

We kindly ask you to complete the table attached. The questionnaire contains questions about the current state of the NBS program in your country, diseases included in the program, plans for the future, and the main obstacles for implementing the expanded NBS program if it is not already implemented.

We plan to publish this study's results in the International Journal of Neonatal Screening (IJNS). All respondents will be invited to cooperate as co-authors. Feel free to contact us through e-mail if you have any questions.

If there is a person in your country who has more direct responsibilities or knowledge in this area, please let us know. Ideally, we would like to get one (or in up to two) merged responses from each country, completed by clinician and laboratory geneticist, responsible for the newborn screening program.

Please complete the table attached by 20th of April 2025. By completing and returning the survey, you give consent for your reply to be incorporated in the study.

Thank you for your participation.

Sincerely,

Urh Grošelj, MD, PhD (urh.groselj@kclj.si)

Nika Požun, MSc

Daša Perko, PhD

Questionnaire: Current Status of Newborn Screening in Southeastern and Central Europe.

Table 1: ***National Data Collection.***

Country	What was the total population in 2024 (in millions)?	What was the GDP per capita in 2024 (USD and EUR)?	How many newborns were born in 2024?	How many newborns were screened in 2024?	How many screening centers were there in 2024?	At what age (hours) are newborns typically screened?
	Click or tap here to enter text.	Click or tap here to enter text.	Click or tap here to enter text.	Click or tap here to enter text.	Click or tap here to enter text.	Click or tap here to enter text.

Table 2: ***Laboratory Methods and NBSP Organization.***

**Country**	**Which laboratory methods are used in the newborn screening program (NBSP)?** *Please check the box next to the answer you choose. Multiple choices are possible.*	**What is the cost of NBSP per newborn (EUR)?**	**How is the NBSP organized?** *Please check the box next to the answer you choose.*
	☐ Fluorometric method<br>☐ Immunofluorescence (DELFIA - Immunoassays included)<br>☐ High-performance liquid chromatography (HPLC)<br>☐ TMS (MS/MS) - Tandem mass spectrometry<br>☐ Radioimmunoassay<br>☐ qPCR<br>☐ Other (please specify): Click or tap here to enter text.	Click or tap here to enter text.	☐ Country-wide organization of NBSP.<br>☐ Regional organization of NBSP.<br>☐ Other (please specify): Click or tap here to enter text.

Table 3: ***Second-tier screening.***

Country	Do you perform second-tier screening in your laboratory?<br>*Please check the box next to the answer you choose.*	
	☐ Yes (including outsourcing)<br>☐ No<br><br>**If you have answered 'Yes', which method do you use, and please specify which disease you use it for?**<br>*Please check the box next to the answer you choose. Multiple choices are possible.*	
	**Method**	**Disease**
	☐ HPLC - High-performance liquid chromatography	Click or tap here to enter text.
	☐ ARMS - Amplification refractory mutation system	Click or tap here to enter text.
	☐ Immunofluorescence	Click or tap here to enter text.
	☐ NGS - Next-generation sequencing	Click or tap here to enter text.
	☐ Sanger	Click or tap here to enter text.
	☐ MLPA - Multiplex Ligation-dependent Probe	Click or tap here to enter text.
	☐ Other (Please provide details on the method and disease): Click or tap here to enter text.	

Table 4: ***Confirmatory methods.***

Country	Do you use genetics as a confirmatory method?<br>*Please check the box next to the answer you choose.*		
	☐ Yes (including outsourcing)<br>☐ No<br><br>**If you answered 'Yes,' which method do you use? Please specify if you do the confirmation on-site or outsource it**.<br>*Please check the box next to the answer you choose. Multiple choices are possible.*		
	**Method**	**On-Site Confirmation**	**Outsourced Confirmation**
	☐ Sanger	☐	☐
	☐ NGS gene panel	☐	☐
	☐ NGS whole exome	☐	☐
	☐ NGS whole genome	☐	☐
	☐ MLPA - Multiplex Ligation-dependent Probe Amplification	☐	☐
	☐ Other (Please provide details on the method and confirmation): Click or tap here to enter text.		

Table 5: ***NBS funding.***

**Country**	**How is the NBSP financed?**<br>*Please check the box next to the answer you choose.*
	☐ By the Ministry of Health<br>☐ Through the national health insurance schemes.<br>☐ Other (please specify): Click or tap here to enter text.

Questionnaire: Current Status of Newborn Screening in Southeastern and Central Europe.

Table 6: ***NBS Screening Diseases.***

Country	Which diseases are mandatory to be screened for, and in which year were they introduced?<br>*In the table below, diseases reported in the previous questionnaire* ***are bolded and highlighted in blue.*** *For diseases introduced between 2019 and 2024, please check the box next to the relevant diseases and write the year of introduction next to each selected disease.*	
	**Disease**	**Year of Introduction**
	☐ ARG - Arginase deficiency	Click or tap here to enter text.
	☐ BTD - Biotinidase deficiency	Click or tap here to enter text.
	☐ CAH - Congenital adrenal hyperplasia	
	☐ CH - Congenital hypothyroidism	Click or tap here to enter text.
	☐ CF - Cystic fibrosis	Click or tap here to enter text.
	☐ CIT1- Citrullinemia type 1	Click or tap here to enter text.
	☐ CIT2 - Citrullinemia type 2	Click or tap here to enter text.
	☐ CPT1 - Carnitine palmitoyltransferase deficiency type 1	Click or tap here to enter text.
	☐ CPT2 - Carnitine palmitoyltransferase deficiency type 2	Click or tap here to enter text.
	☐ CUD - Carnitine uptake defect	Click or tap here to enter text.
	☐ CTN1 - Cardiac troponin 1	Click or tap here to enter text.
	☐ G6PD - Glucose-6-phosphate dehydrogenase deficiency	Click or tap here to enter text.
	☐ GA1 - Glutaric acidemia type 1	Click or tap here to enter text.
	☐ GA2 - Glutaric acidemia type 2	Click or tap here to enter text.

Contiunation of Tabel 6

	☐ GALT - Classic galactosemia	Click or tap here to enter text.
	☐ HBP - Haemoglobinopathy	Click or tap here to enter text.
	☐ HCY - Homocystinuria	Click or tap here to enter text.
	☐ H-PHE - Hyperphenylalaninemia	Click or tap here to enter text.
	☐ HPTI - Hypoxanthine-guanine phosphoribosyltransferase deficiency	Click or tap here to enter text.
	☐ HSD - holocarboxylase synthetase deficiency	Click or tap here to enter text.
	☐ IVA - Isovaleric acidemia (IVA) / 2 - Methylbutyrylglycinuria	Click or tap here to enter text.
	☐ LCHADD - Long-chain 3-hydroxyacyl-CoA dehydrogenase deficiency / TFP-Trifunctional protein deficiency	Click or tap here to enter text.
	☐ MAL - Malonic acidemia	Click or tap here to enter text.
	☐ MCADD - Medium-chain acyl-coenzyme A dehydrogenase deficiency	Click or tap here to enter text.
	☐ MET - Hypermethioninemia	Click or tap here to enter text.
	☐ MSUD - Maple syrup urine disease	Click or tap here to enter text.
	☐ NKH - Non-ketotic hyperglycinemia	Click or tap here to enter text.
	☐ PA/MMA–Propionic / methylmalonic aciduria	Click or tap here to enter text.
	☐ PKU – Phenylketonuria	Click or tap here to enter text.
	☐ SCAD – Short-chain acyl-CoA dehydrogenase deficiency	Click or tap here to enter text.
	☐ SCID – Severe combined immunodeficiency	Click or tap here to enter text.

Contiunation of Tabel 6

	☐ SMA – Spinal muscular atrophy	Click or tap here to enter text.
	☐ TYR1 - Tyrosinemia type 1	Click or tap here to enter text.
	☐ VLCADD – Very long-chain acyl-CoA dehydrogenase deficiency	Click or tap here to enter text.
	**C5OH-related diseases**	
	☐ 3HMGA – 3-Hydroxy-3-methylglutaric aciduria	Click or tap here to enter text.
	☐ BKT - β-ketothiolase deficiency	Click or tap here to enter text.
	☐ 3MCC – 3-methylcrotonyl-CoA carboxylase deficiency	Click or tap here to enter text.
	☐ MCD – Multiple carboxylase deficiency / HSD – Holocarboxylase synthetase	Click or tap here to enter text.
	☐ Other (Please provide details on the disease and year of introduction):<br>Click or tap here to enter text.	

Table 7: ***Further expansion.***

**Country**	**Do you have plans for further expansion?**<br>*Please check the box next to the answer you choose.*
	☐ Yes.<br>☐ No.<br><br>**If you have answered 'Yes', please answer the next question.**

Table 8: ***Further expansion.***

**Country**	**Which diseases are included in the further expansion plan, and when will they be introduced?**<br>*Please fill in the table below. Use the full name of the disease and its abbreviation.*	
	**Disease**	**Year of introduction**
	Click or tap here to enter text.	Click or tap here to enter text.
	Click or tap here to enter text.	Click or tap here to enter text.
	Click or tap here to enter text.	Click or tap here to enter text.
	Click or tap here to enter text.	Click or tap here to enter text.
	Click or tap here to enter text.	Click or tap here to enter text.
	Click or tap here to enter text.	Click or tap here to enter text.
	Click or tap here to enter text.	Click or tap here to enter text.
	Click or tap here to enter text.	Click or tap here to enter text.
	Click or tap here to enter text.	Click or tap here to enter text.
	Click or tap here to enter text.	Click or tap here to enter text.
	Click or tap here to enter text.	Click or tap here to enter text.
	Click or tap here to enter text.	Click or tap here to enter text.
	Click or tap here to enter text.	Click or tap here to enter text.
	Click or tap here to enter text.	Click or tap here to enter text.
	Click or tap here to enter text.	Click or tap here to enter text.
	Click or tap here to enter text.	Click or tap here to enter text.

Table 9: ***Further expansion.***

**Country**	**Do you normally conduct a pilot study before proceeding with expansion?**<br>*Please check the box next to the answer you choose.*	**How do you finance pilot studies?**<br>*Please check the box next to the answer you choose. Multiple choices are possible.*
	☐ Yes.<br>☐ No.<br><br>**If you have answered 'Yes', please answer the next question.**	☐ Institutional funding<br>☐ Government funding<br>☐ Industry sponsorship<br>☐ Grant funding<br>☐ Research collaborations<br>☐ Other (please specify): Click or tap here to enter text.

Questionnaire: Current Status of Newborn Screening in Southeastern and Central Europe.

Table 10: ***Further expansion.***

**Country**	**What are the main reasons that prevent further expansion?** *Please check the box next to the answer you choose. Multiple choices are possible.*	**On a scale of 1 to 5, how would you rate the urgency for expanding Newborn Screening (NBS), with 1 being the lowest and 5 being the highest?**
	☐ Lack of financial resources<br>☐ Lack of staff<br>☐ Lack of organization<br>☐ Later management<br>☐ Low incidences<br>☐ Lack of political will<br>☐ Screening for disease not approved by a medical board committee<br>☐ Other (please specify): Click or tap here to enter text.	☐ 1 (lowest urgency)<br>☐ 2<br>☐ 3<br>☐ 4<br>☐ 5 (highest urgency)

*Table 11: **Consent for NBS.***

**Country**	**What kind of consent do you use for NBS?**<br>*Please check the box next to the answer you choose.*	**How do you inform parents about NBS before obtaining consent?**<br>*Please check the box next to the answer you choose.*
	☐ Opt-In Consent (Parents must actively agree to participate.)<br>☐ Opt-Out Consent (Newborns are automatically included unless parents choose to decline.)<br><br>**How is consent managed?**<br>*Please check the box next to the answer you choose.*<br>☐ Oral Consent<br>☐ Written Consent<br>☐ Other (please specify): Click or tap here to enter text.	☐ During a regular appointment with the gynecologist.<br>☐ After birth by the neonatologist.<br>☐ Other (please specify): Click or tap here to enter text.

Table 12: ***Informing the Public about NBS.***

**Country**	**Do you inform the general public about NBS?**<br>*Please check the box next to the answer you choose.*
	☐ Yes.<br>☐ No.<br><br>**If you have answered 'Yes', how do you inform them?**<br>*Please check the box next to the answer you choose. Multiple choices are possible.*<br><br>☐ Website<br>☐ Brochures and leaflets<br>☐ Other (please specify): Click or tap here to enter text.<br><br>**If you have answered 'Website' or/and 'Brochures and leaflets,' please provide the link to the website or additional details. If brochures can be sent as scans, please include relevant information.**<br>Click or tap here to enter text.

Table 13: ***Sample delivery.***

**Country**	**On average, how long does it take for a sample to be transported from the nursery to the NBS center?**<br>*Please check the box next to the answer you choose.*	**Who brings samples to the clinic?**<br><br>*Please check the box next to the answer you choose. Multiple choices are possible.*
	☐ 1 day<br>☐ 2 days<br>☐ 3 days<br>☐ 4 days<br>☐ 5 days<br>☐ 6 days<br>☐ over 6 days or more	☐ Post office/mail carrier<br>☐ Courier service<br>☐ Other (please specify): Click or tap here to enter text.

Questionnaire: Current Status of Newborn Screening in Southeastern and Central Europe.

Table 14: ***Long-term storage sample.***

Country	**Do you retain samples after analysis for long-term storage?**<br>*Please check the box next to the answer you choose.*
	☐ Yes<br>☐ No<br><br>**If you answered "Yes," please answer the following questions.**<br><br>**1. Please specify how long you retain samples after analysis for long-term storage.**<br>Click or tap here to enter text.<br><br>**2. Where are the samples stored?**<br>☐ Secure archive within the institution<br>☐ Secure archive outside the institution<br>☐ Other (please specify): Click or tap here to enter text.

Contiunation of Tabel 14

	**3. At what temperature are samples stored?**<br>☐ Room temperature (e.g., 15°C-25°C)<br>☐ Refrigerated temperature (e.g., 2°C-8°C)<br>☐ Freezer temperature (e.g.,-20°C or -80°C)<br>☐ Temperature not controlled<br>☐ Other (please specify): Click or tap here to enter text.<br>**4. What is the humidity level in the storage area?**<br>☐ Less than 50%<br>☐ 50%-70%<br>☐ More than 70%<br>☐ Humidity not controlled<br>☐ Other (please specify): Click or tap here to enter text.<br>**5. What light levels are maintained in the storage area?**<br>☐ No light exposure (samples stored in the dark)<br>☐ Limited light exposure (e.g., samples stored in opaque containers)<br>☐ Light not controlled<br>☐ Other (please specify): Click or tap here to enter text.
